# Biological and Demographic Profile of Meningiomas in a Cohort of Egyptian Patients: Impact on Tumor Recurrence

**DOI:** 10.1155/2013/375139

**Published:** 2013-12-26

**Authors:** Eman Abdelzaher, Nevine M. F. El Deeb, Ahmed G. Gowil, Ahmed Yehya

**Affiliations:** ^1^Department of Pathology, Faculty of Medicine, University of Alexandria, Alexandria 21533, Egypt; ^2^Department of Oncology and Nuclear Medicine, Faculty of Medicine, University of Alexandria, Alexandria 21533, Egypt; ^3^Department of Neurosurgery, Faculty of Medicine, University of Alexandria, Alexandria 21533, Egypt

## Abstract

*Objective*. This work was designed to study the biological and demographic characteristics of meningiomas and their impact on tumor recurrence in Egyptian patients. *Material and Methods*. A cohort of 265 Egyptian patients with meningioma was studied. Immunohistochemistry for VEGF, Ki67, PR, CD20, and CD3 was performed. Statistical analysis was used to detect independent predictors of recurrence. *Results*. Adults represented 98.9% of cases, with female preponderance (M : F ratio = 1 : 2.4). Histologically, 78.10% of cases were grade I, 19.20% were grade II, and 2.60% were grade III. Transitional variant was the most common (43.40%). VEGF expression (38.50% of cases) correlated positively with perifocal edema, tumor size, and proliferative index (PI). PR expression (64.5% of cases) correlated inversely with the PI (mean 3.75). Lymphocytic aggregates were detected in 7.20% of cases, with a mean CD20 : CD3 ratio of 1 : 10.1. In a multivariate analysis, only tumor size, PR expression and necrosis predicted recurrence independently. Using ROC curve, size was the best predictor of tumor recurrence with a cut-off point of >6 cm and an excellent negative predictive value (97.6%). *Conclusions*. Meningiomas in our region showed some distinctive clinicopathological and demographic criteria. Tumor size was found to be the best recurrence predictor factor of meningioma.

## 1. Introduction

Meningiomas, deriving from meningothelial (arachnoid cap) cells, are the most common primary intracranial and spinal intradural neoplasms [1].

Despite their prevalence among central nervous system (CNS) tumors, their epidemiology, biological behavior, and clinical outcomes have been poorly defined. This has been attributed to the lack of uniform database registration [2].

Characterizing meningiomas, with respect to their demographic and biological features, in different regions of the world may provide clues to meningioma etiology and behavior. It also helps planning clinical and basic research protocols, serves as a major guide to novel therapeutic technologies, and allows evaluation of the medical practices and standardization of healthcare services [3, 4].

Most meningiomas pursue a benign course; despite this, patients still experience tumor recurrence. Tumor grade and extent of resection remain the most reliable predictors of meningiomas' behavior. However, more studies from different regions of the world are required to investigate other predictors of recurrence [5, 6].

This work was designed to study the biological and demographic characteristics of meningiomas and their impact on tumor recurrence in a cohort of Egyptian patients.

## 2. Materials and Methods

### 2.1. Patients and Tissue Samples

The present work was conducted on 265 retrospective meningioma cases (from 2004 to 2012). The patients reside in Alexandria and Beheira governorates, Egypt. Clinical, neuroimaging, operative, and follow-up data were available for all patients. Follow-up included immediate postoperative CT, which was followed by regular visits that included clinical and neurological examination as well as CT imaging.

Histopathological typing and grading were done according to WHO criteria [7]. Ectopic meningiomas were excluded from the study.

### 2.2. Immunohistochemistry

Tissue macroarray blocks were constructed as previously described [8, 9]. A hematoxylin and eosin stained section of each tissue macroarray block was first examined to ensure representative selection for the histological type and grade of meningioma. Other sections were mounted on positively charged slides for immunohistochemical studies.

Immunohistochemical staining was performed using an avidin-biotinylated immunoperoxidase methodology. The used primary antibodies (at 1 : 100 dilution): vascular endothelial growth factor (VEGF), clone: VG; Ki67, clone: SP6; progesterone receptor (PR), clone: SP2; CD20 Ab-1, clone: L26; and CD3epsilon Ab-2, clone: PS1, as well as the detection kit (UltraVision Detection System Anti-Polyvalent, HRP/DAB, Ready-To-Use), were purchased from Thermo Scientific Lab Vision, USA. VEGF, CD20, and CD3 were mouse monoclonal antibodies while Ki67 and PR were rabbit monoclonal antibodies. Positive and negative controls were included in all runs.

### 2.3. Evaluation of Immunohistochemical Staining

For VEGF and PR, immunostained sections were graded semiquantitatively for intensity and extent of immunostaining. The staining intensity was scored as follows: 0—no staining; 1—mild staining; 2—moderate staining; and 3—intense staining. The percentage of positive cells was scored as follows: 0—0%; 1—≤25%; 2—>25–≤50%; 3—>50–≤75%; 4—>75%.

The final score was calculated by adding points obtained from the two aforementioned scoring systems: range 0–7 [5].

As for Ki67, areas with the highest density of Ki67-immunostained nuclei were defined, and the Ki67 proliferative index (PI) was expressed as a percentage [5].

T and B lymphocytes were highlighted by CD3 and CD20 immunostains, respectively, and an approximate estimate of T- to B-lymphocyte ratio was performed [10].

### 2.4. Statistical Analysis

Statistical analyses were performed using SPSS Statistics 20. Correlation between different biomarkers was conducted using Spearman's correlation and Mann-Whitney *U* test. Logrank test was used to compare the recurrence distributions between different groups. Bivariate Cox regression was used to evaluate the effect of continuous covariates on tumor recurrence. Factors with a strong bivariate significance indicated by a *P* value below 0.05 were included in a multivariate Cox regression model. Multicollinearity among independent variables was tested using variance inflation factor. Receiver operator characteristic (ROC) was used to judge the prognostic performance of different markers and cut-off points were determined using Youden's index. Significance was judged at the 5% level.

## 3. Results

### 3.1. Clinicopathological Findings

The age of the patients ranged from 12 to 80 years (median = 50; mean = 50.81, SD = 12.24). Elderly patients (≥70 years) constituted 6.8% of the cases. Pediatric cases (≤18 years) were a minority (3 cases, 1.13%). The male to female ratio was 1 : 2.4. In grade II and III lesions, the male to female ratio was 1 : 1.

Nearly half of the patients (48.30%) presented with generalized manifestations of increased intracranial pressure. The rest of the patients (51.70%) presented with localizing symptoms. A single case (0.40%) was associated with neurofibromatosis type 2 (NF2).

Neuroimaging and operative data showed that cranial meningiomas (88.30%) predominated over spinal tumors. Supratentorial cranial location (80.40%) was the most common with cerebral convexities being the most frequently affected site (46.00%) (Table 1). Elderly patients showed the same tendency for cranial predilection (88.88%). Basal meningiomas constituted 37.20% of the cases.

Spinal meningiomas (11.70%) were confined to dorsal (8.70%) and cervical (3.00%) regions with a ratio of 2.9 : 1. They were all grade I with a male to female ratio of 1 : 6.8.

Overall, the right and left sides were nearly equally affected with a right to left ratio of 1.1 : 1.

The size ranged from 2 to 9 cm (median = 4, mean = 4.44, SD = 1.66). Perifocal edema was encountered in 37.7% of cases and ranged in extent from mild to severe with mild edema being the most prevalent (18.50%). A significant positive correlation was found between tumor size and perifocal edema (*ρ* = 0.344, *P* = 0.000).

Macrocysts were detected in 8 cases (3.00%). Hyperostosis was noted in 13.60% of cases. Four patients (1.50%) presented with multiple tumors.

Nearly two-thirds of the patients (66.80%) underwent gross total surgical resection of the tumors (Simpson's grades I and II); and one-third of the patients (33.20%) underwent subtotal resection (Simpson's grades III and IV). The extent of surgical resection was confirmed by immediate postoperative CT. Postoperative radiotherapy followed for patients with subtotal resection as well as all grade II and III tumors.

Histologically, 207 cases (78.10%) were grade I, 51 (19.20%) were grade II, and 7 (2.60%) were grade III. Overall, the transitional variant was the most common (43.40%) followed by the meningothelial and fibrous variants (18.90% and 15.50%, resp.) (Table 1).

**Figure 1:**
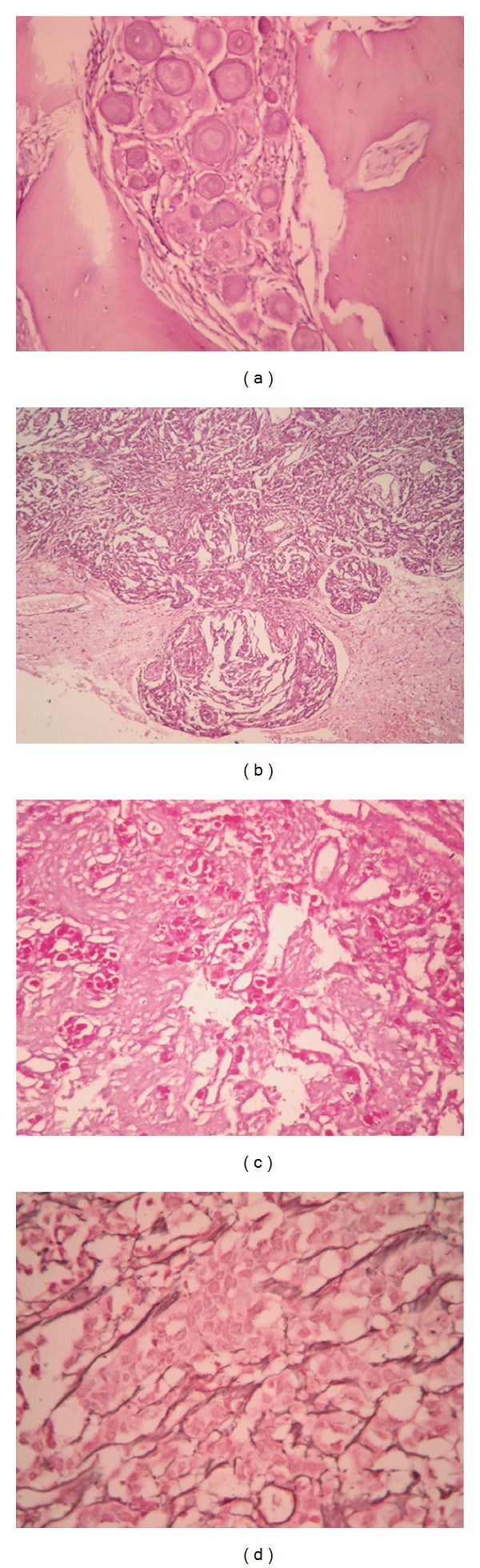
(a) Bone invasive meningioma: an island of meningioma tissue with numerous psammoma bodies is seen invading lamellar bone trabeculae (H&E, 100x); (b) brain invasive meningioma: tongues of meningioma tissue invading brain parenchyma are noted (H&E, 40x); (c) secretory meningioma: PAS histochemical stain is highlighting the intracytoplasmic inclusions characteristic of this subtype (PAS, 100x); (d) reticulin fibers are surrounding syncytial groups of meningioma cells (reticulin, 200x).

Psammoma bodies (excluding psammomatous meningioma) and lymphocytic aggregates were seen in 57.00% and 7.20% of cases, respectively. Bone (Figure 1(a)) and soft tissue invasion were detected in 8.70% and 2.60% of cases respectively. Brain invasion (Figure 1(b)) was seen in 22 (37.93%) out of the 58 cases of grade II and III intracranial tumors. One or two atypical histological features were detected in 54 (26.10%) out of the 207 cases of grade I meningioma. Hypercellularity and nucleolar prominence (11.6% each) were the most common, followed by necrosis (9.7%).

**Figure 2:**
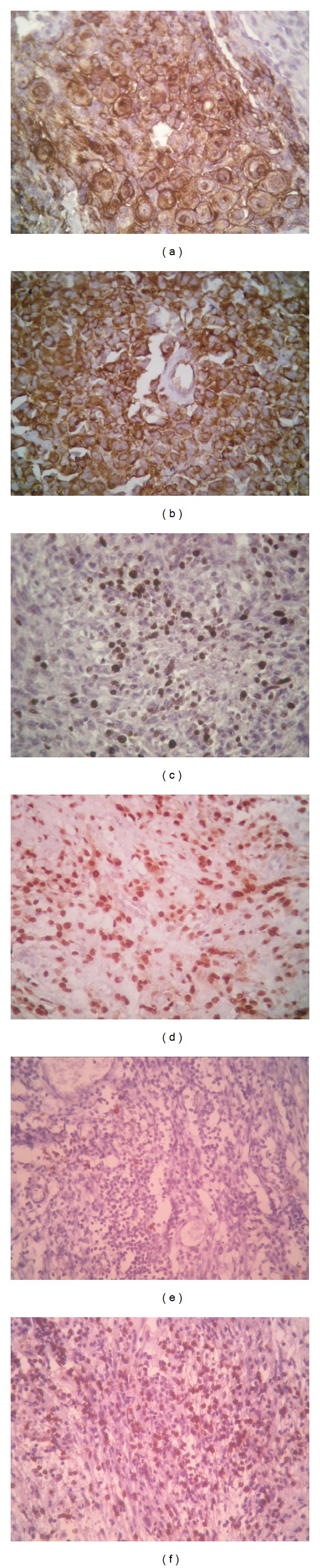
(a) EMA immunostain highlighting whorls of meningioma cells (200x); (b) intense and diffuse VEGF expression in an anaplastic meningioma (200x); (c) Ki67 immunostain revealing a moderate proliferative activity in a case of atypical meningioma (200x); (d) benign meningioma with significant PR expression (200x); (e) CD20 highlighting rare B lymphocytes (100x); (f) CD3 showing a large population of T lymphocytes (100x).

PAS and reticulin histochemical stains were applied whenever needed (Figures 1(c) and 1(d)). Immunohistochemistry for EMA was done to confirm the diagnosis in 84 cases (31.70%) (Figure 2(a)).

During the follow-up period (median = 54 months, range = 2–100 months), 27 patients (10.20%) experienced tumor recurrence (mean recurrence free survival (RFS) = 51.75 months, SD = 26.69) that was radiologically and/or histopathologically proven. Nineteen of the recurrent tumors (70.37%) were grade I, six (22.22%) were grade II, and two (7.41%) were grade III.

Histological data were available for 21 recurrent tumors. Sixteen tumors were of the same grade and five showed progression to a higher grade.

### 3.2. Immunohistochemistry

VEGF positivity was confined to meningioma tumor cells and vascular endothelial cells (Figure 2(b)). It was detected in 102 cases (38.50%) with a median staining score of 3 (range = 2–7).

VEGF expression correlated positively with perifocal edema and tumor size (*ρ* = 0.837, *P* = 0.000 and *ρ* = 0.343, *P* = 0.000, resp.). VEGF expression was not related significantly to cyst formation (*U* = 695.500, *P* = 0.074).

The PI ranged from 0.1 to 25% (median = 2, mean = 3.75, SD = 3.88) (Figure 2(c)).

PR positivity (Figure 2(d)) was demonstrated in 171 cases (64.53%) with a median staining score of 4 (range = 2–6).

VEGF expression was significantly positively correlated with PI (*ρ* = 0.303, *P* = 0.000). VEGF and PR showed a tendency towards an inverse correlation, yet it was statistically insignificant (*ρ* = −0.113,*P* = 0.066). A significant negative correlation was found between PR expression and PI (*ρ* = −0.219,*P* = 0.000).

The CD20 : CD3 ratio ranged from 1 : 3 to 1 : 19 (median = 1 : 9; mean = 1 : 10.1, SD = 6.2) (Figures 2(e) and 2(f)).

## 4. Relationship between Clinicopathological Parameters and Expression of Biomarkers with Tumor Recurrence

In a bivariate analysis, recurrence was significantly associated with the following parameters: tumor grade (*P* < 0.001), tumor size (HR = 1.9; 95% CI = 1.5, 2.5; *P* < 0.001), nucleolar prominence (*P* = 0.044), necrosis (*P* < 0.001), VEGF expression (HR = 1.6; 95% CI = 1.3, 1.9; *P* < 0.001), PR expression (HR = 0.7; 95% CI = 0.6, 0.9; *P* < 0.001), and PI (HR = 1.3; 95% CI = 1.2, 1.4; *P* < 0.001).

In a multivariate analysis using Cox proportional-hazards regression, only tumor size (adjusted HR = 1.8, 95% CI = 1.3,2.5), PR expression (adjusted HR = 0.6, 95% CI = 0.5, 0.8), and necrosis (adjusted HR = 2.7, 95% CI = 1, 7.1) were found to predict recurrence independently. PI was excluded to avoid multicollinearity.

**Figure 3:**
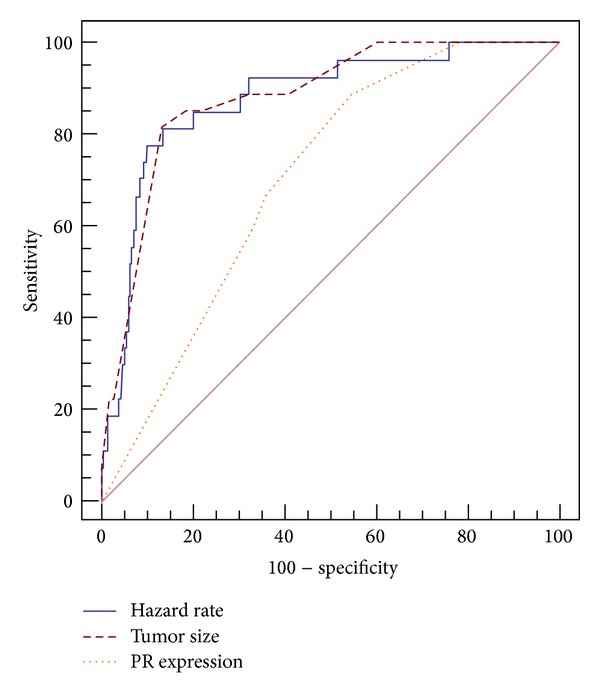
ROC curve—prognostic accuracy of tumor size and PR expression compared to hazard rate among the 265 cases of meningioma studied.

The three independent predictors were further regressed against the dependent variable (“recurrence” with “recurrence free survival”). The prognostic accuracy of the developed model was compared to that of tumor size, PR, and necrosis using ROC curve (Table 2 and Figure 3).

The comparison between predictabilities of tumor size and model revealed insignificant difference (difference area = 0.002, *P* = 0.953). Either of them predicted recurrence better than PR did (difference area = 0.177, *P* < 0.001 and difference area = 0.174, *P* < 0.001, resp.). The sensitivity of necrosis was the lowest (40.74%) and its specificity (83.61%) was lower than hazard rate and size.

Thus, the size was found to be the best predictor of tumor recurrence with a cut off point of >6 cm, a positive predictive value of 41.5%, and an excellent negative predictive value (97.6%).

## 5. Discussion

Meningiomas are among the most common CNS tumors; however, their epidemiological data in Egypt have been rather incomplete except for some regional reports [4].

There are no available demographic data on meningiomas in our region (Alexandria and Beheira governorates, Egypt).

The present work showed that the studied cohort of meningioma patients had some demographic and clinicopathological criteria in common with meningioma patients from other countries, including predominant affection of adult female patients with supratentorial cerebral convexities being the most common site [2, 11, 12]; rarity in pediatric population [13]; infrequency of macrocysts [14]; predominance of benign grade I histology [2, 11, 12]; frequency of basal meningiomas [15]; and frequency of spinal meningiomas and their higher predilection for females, dorsal spine affection, and predominant benign histology [16].

Compared to published reports from other countries [13, 17–24], our study has demonstrated some demographic and clinicopathological differences in meningiomas in our area, including the lack of male preponderance in atypical/malignant meningiomas and their near twofold higher incidence compared to that cited in the literature; the lower incidence of meningiomas in elderly patients and its higher cranial predilection; the rarity of multiple lesions and NF2-associated lesions; the lower incidence of hyperostosis; and the smaller average tumor size, as well as the predominance of transitional over meningothelial histology.

Other demographic and clinicopathological characteristics that have not been widely studied previously [5, 25] were documented in our work including the near equal right/left side affection; the frequency of psammoma body occurrence in nonpsammomatous meningiomas; bone and soft tissue invasion; and brain invasion, as well as the frequency of atypical histological features in benign meningiomas and the incidence of lymphocytic infiltration.

Despite the fact that a large majority of meningiomas are classified as benign lesions, there is great heterogeneity in histology, aggressiveness, recurrence rates, and survival outcomes [2].

In the present series, 27 patients (10.2%) experienced tumor recurrence which is in agreement with previous reports [26].

The expression of established and newly emerging prognostic biomarkers was investigated in this study in order to evaluate their impact, together with other clinicopathological variables, on tumor recurrence.

The used “tissue macroarray” methodology is a powerful, dependable, and cost-effective way for simultaneous analysis of multiple specimens with immunohistochemistry. This method avoids the disadvantages of laborious methods of tissue arraying, such as expensive equipment and scarce tissue sampling, and it can be implemented in any institution with minimal cost and elaboration. Thus, it has been recommended as an alternative efficient approach for large-scale studies [8, 9].

VEGF and its receptors are known to be frequently expressed in meningiomas and seem important for tumor growth and recurrence [27].

Understanding the role of VEGF expression in meningiomas might contribute to the development of a new therapeutic strategy.

In this study, VEGF expression in meningiomas was found to be less frequent than that in previous reports [12, 27, 28] and it was localized to both tumor and vascular endothelial cells as previously reported [28].

The demonstrated significant positive correlation between VEGF and PI and the insignificant relation with PR expression are in accordance with previous studies [27, 29].

Perifocal edema in meningiomas has been recognized as a common problem which can increase the mass effect of the tumor, aggravate clinical symptoms, raise intracranial pressure, and affect tumor management [30].

Our findings are in agreement with previous reports documenting that approximately 40–60% of patients with meningiomas have perifocal edema, mostly in a mild form [30–32].

This study showed a significant relation between VEGF expression and perifocal edema which together with previous reports implicates VEGF in the pathogenesis of meningioma-related edema [12, 28, 33, 34]. Therefore, VEGF-targeted therapy may represent a novel therapeutic approach in meningioma management.

Despite the significant relationship between VEGF expression and edema formation in our study, VEGF was not related to macrocyst formation, a finding that contrasts with previous reports [35].

In meningiomas, proliferation rates and PR status are regarded as useful biologic indicators of tumor activity [18].

In this work, the demonstrated high percent of PR expression and its inverse correlation with PI as well as the mean PI in the studied group conform to previous reports [18, 36, 37].

The study of immune cells in meningiomas has not received much attention previously and contradicting results have been reported [25, 38].

In our study, lymphocytic infiltrates were encountered in 7.20% of the cases. The T cells predominated (mean CD20 : CD3 ratio = 1 : 10.1) which is in agreement with previous reports [38].

The natural history of surgically treated meningiomas can be quite variable. Recurrence and patient outcome cannot currently be predicted with accuracy [39].

In this study, a bivariate analysis showed that recurrence was significantly associated with tumor size, tumor grade, nucleolar prominence, necrosis, VEGF expression, PR expression, and PI.

However, in a multivariate analysis, only size, PR expression, and necrosis were found to predict recurrence independently. Comparing the three parameters, the size was found to be the best recurrence predictor factor with a cut-off point of >6 cm and an excellent negative predictive value (97.6%).

Tuna et al. [40] have labeled meningiomas larger than 6 cm “huge meningiomas” and found that these tumors had less favorable prognosis.

Chan and Thompson [41] reported that patients with meningiomas measuring 4.5 cm or less in diameter had a significantly better prognosis than did those with tumors measuring 7 cm or more.

The adverse effect of size could be attributed to its effect on the extent of surgical removal (Simpson's grade), which is an important prognostic factor and affects operative morbidity and mortality [40, 42, 43].

In our study, tumor size correlated positively with VEGF expression and perifocal edema pointing to their potential negative impact on prognosis that needs further investigation.

To the best of our knowledge, this is the first study that specifically addresses the issue of demographic and biological characteristics of meningiomas in a large cohort of Egyptian patients and their impact on tumor recurrence.

In conclusion, the present study shed light on the demographic and biological characteristics of meningioma in our locals. The studied meningiomas have general demographic and clinicopathological profile in common to that reported from other countries with some interesting differences.

In a multivariate analysis, including clinicopathological factors and expression of recent and established biomarkers, only tumor size was found to be the best recurrence predictor factor with a cut-off point of >6 cm and an excellent negative predictive value (97.6%).

Thus, tumor size greater than 6 cm can identify meningiomas with a high risk of recurrence, which could be beneficial for planning tailored optimal surgical and follow-up strategies.

## Figures and Tables

**Table 1:** Site distribution and histopathological types of the 265 cases of meningioma studied.

		No. of cases	(%)
Site	Convexity	122	(46.00)
	Spinal	31	(11.70)
	Anterior cranial fossa	23	(8.70)
	Sellar/suprasellar	21	(7.90)
	Posterior fossa	19	(7.20)
	Sphenoidal	18	(6.80)
	Falcine/parasagittal	17	(6.40)
	Middle cranial fossa	7	(2.60)
	lateral ventricular	4	(1.50)
	Miscellaneous	3	(1.10)

Histopathological type	Transitional	115	(43.40)
	Meningothelial	50	(18.90)
	Fibrous	41	(15.50)
	Psammomatous	20	(7.50)
	Microcystic	13	(4.90)
	Angiomatous	8	(3.00)
	Secretory	6	(2.30)
	Anaplastic	6	(2.30)
	Chordoid	4	(1.50)
	Metaplastic	1	(0.40)
	Papillary and rhabdoid	1	(0.40)

**Table 2:** Prognostic accuracy of tumor size, PR expression, and necrosis compared to hazard rate among the 265 cases of meningioma studied.

Prognostic marker	AUC (95%CI)	*P* value	Cut-off value	Sn (95% CI)	Sp (95% CI)	+LR (95% CI)	−LR (95% CI)	PPV (95% CI)	NPV (95% CI)
Hazard rate from the model	0.878 (0.832 to 0.914)	<0.001	>0.0926	81.48 (61.9–93.7)	86.55 (81.6–90.6)	6.06 (4.2–8.8)	0.21 (0.10–0.5)	40.7 (27.4–55.1)	97.6 (94.6–99.2)
Tumor size	0.880 (0.835 to 0.917)	<0.001	>6	81.48 (61.9–93.7)	86.97 (82.0–91.0)	6.26 (4.3–9.1)	0.21 (0.10–0.5)	41.5 (28.0–56.0)	97.6 (94.6–99.2)
PR expression	0.703 (0.644 to 0.758)	<0.001	≤3	88.89 (70.8–97.6)	45.38 (38.9–51.9)	1.63 (1.4–1.9)	0.24 (0.08–0.7)	15.6 (10.2–22.3)	97.3 (92.3–99.4)
Necrosis*				40.74 (22.4–61.2)	83.61 (78.3–88.1)	2.49 (1.5–4.3)	0.71 (0.5–1.0)	22.0 (11.4–36.1)	92.6 (88.2–95.7)

*ROC curve analysis was not performed for necrosis as it is a dichotomous variable.

AUC: area under the curve; Sn: sensitivity; Sp: specificity; +LR: positive likelihood ratio; −LR: negative likelihood ratio; PPV: positive predictive value; NPV: negative predictive value.
